# The Missing LNK: Evolution from Cytosis to Chronic Myelomonocytic Leukemia in a Patient with Multiple Sclerosis and Germline SH2B3 Mutation

**DOI:** 10.1155/2022/6977041

**Published:** 2022-03-01

**Authors:** Krishna Gundabolu, Bhavana J. Dave, Carmelita J. Alvares, Jeffrey J. Cannatella, Vijaya R. Bhatt, Lori J. Maness, Zaid S. Al-Kadhimi, Rana K. Zabad, Allison M. Cushman-Vokoun

**Affiliations:** ^1^The Fred and Pamela Buffett Cancer Center, Department of Internal Medicine, Division of Hematology-Oncology, University of Nebraska Medical Center, Omaha, NE, USA; ^2^Department of Pathology and Microbiology, University of Nebraska Medical Center, Omaha, NE, USA; ^3^Munroe-Meyer Institute for Genetics and Rehabilitation, University of Nebraska Medical Center, Omaha, NE, USA; ^4^Department of Neurological Sciences, University of Nebraska Medical Center, Omaha, NE, USA

## Abstract

Chronic myelomonocytic leukemia (CMML) is a rare but distinct hematological neoplasm with overlapping features of myelodysplastic syndrome (MDS) and myeloproliferative neoplasm (MPN). Individuals with CMML have persistent monocytosis and bone marrow dyspoiesis associated with various constitutional symptoms like fevers, unintentional weight loss, or night sweats. It is established that there is a strong association of CMML with preceding or coexisting autoimmune diseases and systemic inflammatory syndromes affecting around 20% of patients. Various molecular abnormalities like *TET2, SRSF2, ASXL1,* and *RAS* are reported in the pathogenesis of CMML, but no such mutations have been described to explain the strong association of autoimmune diseases and severe inflammatory phenotype seen in CMML. Germline mutation in SH2B adaptor protein 3 (*SH2B3*) had been reported before to affect a family with autoimmune disorders and acute lymphoblastic leukemia. In this report, we describe the first case of a female subject with many years of preceding history of multiple sclerosis before the diagnosis of CMML. We outline the evidence supporting the pathogenic role of *SH2B3 p.E395K* germline mutation, connecting the dots of association between autoimmune diseases and CMML genesis.

## 1. Introduction

Genes influencing epigenetics, cell signaling, splicing machinery, and transcription factors of the hematopoietic cells are commonly mutated in chronic myelomonocytic leukemia (CMML) [1]. The Janus-activated kinase/signal transducer and activator of transcription (JAK/STAT) pathway plays a key role in normal hematopoietic stem cell growth and differentiation and is activated abnormally in various myeloproliferative neoplasms (MPNs), including CMML. In normal circumstances, various growth factors (erythropoietin, thrombopoietin, granulocyte-macrophage colony stimulating factor) and cytokines (like IL-3 and IL-7) activate this pathway, and negative regulators of this pathway like SOCS1/3 and LNK adaptor protein are crucial in limiting cell proliferation. LNK (encoded by the gene *SH2B3*) contains an important src homology 2 (SH2) domain that adapts to phosphorylated JAK2, thereby negatively regulating JAK2-activated pathways [2]. Germline mutations in the *LNK*/SH2B adaptor protein 3 (*SH2B3*) gene have been reported in acute lymphoblastic leukemia and MPNs other than CMML [3, 4], and various single-nucleotide polymorphisms are associated with autoimmune diseases, adipose tissue inflammation, diabetes, and cardiovascular diseases [5–7]. While somatic mutations are well described in the pathogenesis of CMML, germline mutations outside of syndromes such as RASopathies are not well characterized. We report the first case of CMML with *SH2B3* p.E395K germline mutation.

## 2. Case Presentation

**Figure 1:**
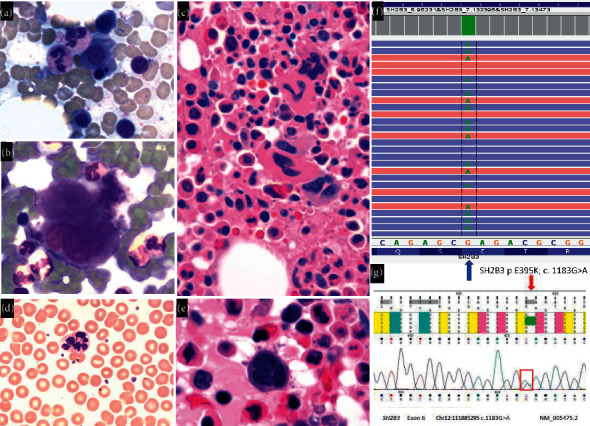
(a) Erythroid nuclear dyskinesis (bone marrow aspirate, 600x magnification Wright-Giemsa staining). (b) Megakaryocyte with unremarkable nuclear lobes (bone marrow aspirate smear, 500x magnification; Wright-Giemsa staining). (c) Hypercellular marrow with focal megakaryocyte clustering (core biopsy, 500x magnification; Hematoxylin and Eosin staining). (d) Hypersegmented neutrophil (peripheral blood, 600x magnification; Hematoxylin and Eosin staining). (e) Hypercellular marrow with micro-megakaryocyte (core biopsy, 1000x magnification, Hematoxylin and Eosin staining). (f) The SH2B3 p.E395K; c 1183G > A mutation identified by NGS as shown in the Integrative Genomics Viewer (IGV) (Broad Institute, Cambridge MA). The blue and red bars represent reverse and forward reads, respectively, and the bars with green As are mutated reads. This image demonstrates the mutation from DNA extracted from the follow-up bone marrow specimen. (g) The *SH2B3* c.1183G > A (p.E395K) heterozygous missense variant detected in the targeted sequencing study (RefSeq NM_005475.2) by direct sequence analysis utilizing automated fluorescence dideoxy sequencing. The red arrow and box demonstrate the missense variant region observed in the genomic DNA extracted from the cultured skin fibroblasts.

A 37-year-old Caucasian woman was diagnosed with multiple sclerosis (MS) after presenting with headaches, optic neuropathy, and peripheral vision loss. A complete blood count at the time of diagnosis of MS showed leukocytosis and thrombocytosis (Supplementary Materials (see here)). A bone marrow biopsy performed 20 months from diagnosis of MS showed hypercellular marrow (80%) with orderly trilineage hematopoietic maturation and mild erythroid dyspoiesis (Figures 1(a)–1(c)). No anomalies were detected by flow cytometry. Cytogenetic studies showed a normal diploid karyotype, and no abnormalities were identified by a myelodysplastic syndrome (MDS)/MPN fluorescence in situ hybridization (FISH) panel. *JAK2* p.V617F mutation analysis by next-generation sequencing (NGS) and BCR/ABL1 qualitative screening by reverse transcriptase polymerase chain reaction (RT-PCR) were unremarkable. A 40-gene myeloid mutation panel, validated for somatic mutation analysis using semiconductor-based NGS (Ion Torrent, ThermoFisher Scientific™, Waltham, Massachusetts), was performed on genomic DNA extracted from a peripheral blood specimen. This assay identified a variant in *SH2B3* (NCBI Accession NM_005475.2) in exon 6 (p.E395K; c.1183G > A; sequence compared to hg19 reference sequence) with a variant allele frequency (VAF) of 52%. It was classified as likely pathogenic due to its presence in the conserved src homology 2 (SH2) domain, the association of *SH2B3* variants with MPNs, and recent data indicating that the variant was pathogenic in the germline setting of familial erythrocytosis [8]. Other established driver mutations in genes that are associated with various MPNs, including those in *JAK2* (exons 12–15), TPO receptor (MPL), and calreticulin (CALR) were not detected. Overall, the bone marrow morphological and immunophenotypic evaluation were insufficient to establish a diagnosis of MPN.

Because of the VAF of *SH2B3* at 52% in this patient, and because there is a population frequency associated with this SH2B3 variant in the gnomAD browser (Broad Institute, Cambridge, MA) of 0.015%, it was considered that this could potentially be a germline variant. Therefore, after consent, a fibroblast culture was established from a skin punch biopsy and DNA was extracted. A targeted dideoxy sequencing assay was performed, detecting the same *SH2B3* heterozygous missense variant, indicating that it was of germline origin (Figure 1(g)) classified as a variant of unknown clinical significance using germline classification guidelines. Outside breast cancer, there was no significant family history of hematological disorders at the time of presentation and genotyping of family members could not be performed. Using the DNA extracted from the patient's skin fibroblasts, a customized 83-gene NGS panel performed for looking into hereditary cancer syndromes revealed heterozygous variants of uncertain significance (based on the NCBI ClinVar database) in exon 22, PDGFRA (c.2989G > A (p.E997K): NM_006206.4 cDNA reference sequence) gene, and exon 1, VHL (c.25G > A (p.D9N): NM_000551.3 cDNA reference sequence) gene. Screening for Fanconi anemia by chromosome breakage using mitomycin C or diepoxybutane was unremarkable.

The patient subsequently developed increasing leukocytosis and frequent night sweats. A repeat bone marrow biopsy was performed 2 years since the original one, which showed a hypercellular marrow (90%) with dysgranulopoiesis, dysmegakaryopoiesis, and persistent monocytosis, consistent with CMML without excess blasts (CMML-0; Figures 1(d) and 1(e)). Flow cytometry was unremarkable. Cytogenetic studies by karyotype analysis and a *FISH* panel for *MDS/MPN* showed normal results including proves for *JAK2*, *PDGFRA*, *PDGFRB*, and *FGFR1* gene rearrangements. A myeloid mutation panel was performed on DNA extracted from the repeat bone marrow, which demonstrated the *SH2B3* p.E395K variant at 51% VAF (Figure 1(f)). As two years had passed, this variant was subsequently classified as pathogenic by one submitter in the NCBI ClinVar database and was also modeled in silico by PolyPhen and SIFT programs as probably damaging and deleterious, respectively. Because of these updates, and due to the patient's clinical progression, the classification of ”likely pathogenic” was maintained from the original mutation analysis, assessing for somatic mutations. No other somatic mutations were identified upon the subsequent analysis of the bone marrow.

## 3. Discussion

CMML is a type of MDS/MPN overlap neoplasm with clinical, laboratory, and morphological features suggestive of both MDS and MPN. Various somatic mutations have been reported with great frequency in CMML with *TET2* (∼50–60%), *SRSF2* (∼50%), *ASXL1* (∼40%), *SETBP1* (∼15%), and RAS pathway (∼30% involving *KRAS*, *NRAS*, *PTPN11*, *CBL*, and *NF1*) being the most frequent of all [9]. Somatic mutations in *SH2B3* are rare in myeloid neoplasms, and only a handful cases of germline *SH2B3* mutations have been reported so far in patients with MDS/MPN, who had various clinical phenotypes based on the coexisting somatic driver mutation (Table 1) [10–13]. In addition to MPNs, *SH2B3* mutations have also been discovered in people with idiopathic erythrocytosis without a clear etiology and with subnormal erythropoietin.

**Figure 2:**
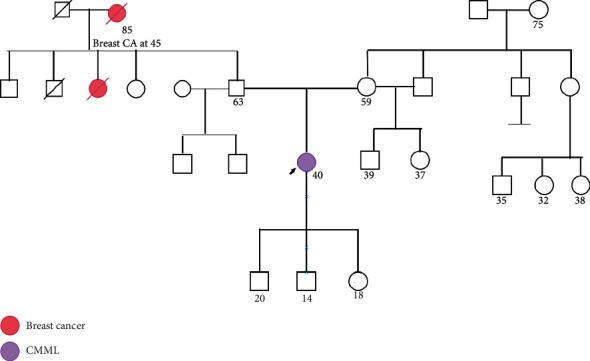
Pedigree chart of the family.

The *SH2B3* gene encodes for an adaptor protein, LNK, which is a negative regulator of cytokine signaling through interaction between its SH2 domain and phosphorylated *JAK2,* attenuating JAK2/STAT signaling. This negative feedback loop is important for immunologic balancing and regulation in physiological and pathological conditions in which the JAK/STAT pathway is activated, such as MPNs. The *SH2B3* gene has 8 exons and is located on chromosome 12q24. There are three functional domains in LNK which includes Phenylalanine Zipper (dimerization domain), Pleckstrin Homology (PH), and Src Homology 2 regulatory (SH2) domains [4]. Various somatic mutations affecting any one of those domains have been described so far in patients with MPNs. The germline mutations reported so far includes p.E208Q and p.E400K which affects PH and SH2 domains, respectively. Our patient's mutation leads to substitution of lysine for a glutamate at position 395, which substitutes a positively charged amino acid (Lys, K) for a negatively charged amino acid (Glu, E). This change in charge could potentially affect the SH2 domain function. The importance of the heterozygous variants discovered in PDGFRA and VHL genes is uncertain as she did not have any personal or family history of gastrointestinal stromal tumors seen with PDGFRA germline mutations, renal cell carcinoma, pheochromocytoma, or other tumors seen in von Hipple–Lindau (VHL) disease germline mutation (Figure 2).

Patients with CMML have increased odds of preceding history of chronic inflammation and/or autoimmune diseases [14]. The mechanistic link behind that is still unclear. Mutations in *SH2B3* are strongly linked with inflammatory and autoimmune syndromes, and the locus 12q24 has been postulated to be linked to type 1 diabetes mellitus [15]. Our patient had a history of multiple sclerosis preceding the diagnosis of CMML, suggesting that the *SH2B3* mutation is possibly associated with both conditions. She continues to receive her treatment of MS with ocrelizumab and is currently on aspirin without any cytoreductive therapy for CMML.

## 4. Conclusions

This case uniquely shows the evolution of abnormal hematopoiesis in a patient with a history of multiple sclerosis and a germline *LNK/SH2B3* p.E395K mutation, who developed progressive changes in bone marrow morphology in tandem with the evolution of cytosis to CMML. Interestingly, no other common driver mutations were identified in this patient, at least in the 40 genes assessed by the NGS panel, which includes all genes mutated in CMML at a high frequency. This report also suggests that dysregulation of LNK/SH2B3 can be one of the possible reasons behind the pathogenesis of the observed strong association between autoimmune diseases and CMML, which needs further exploration. Finally, this case demonstrates that classification of rare variants is difficult, especially when the somatic versus germline nature of the variant is not known. If there is any suspicion that a variant, identified by somatic mutation analysis, could be a germline mutation, further discussion regarding fibroblast culture analysis and genetic counseling may be appropriate in the setting of a hematologic disorder.

## Figures and Tables

**Table 1:** Germline mutations in SH2B3, previously reported in MPN.

Germline mutation (domain)	Disease	Driver mutation	Reference
LNK p.E395K (SH2)	CMML	Not identified	Present case
LNK p.E400K (SH2)	MDS/MPN-RS-T	SF3B1	[10]
LNK p.E208Q (PH)	PMF	CALR type 1 [11]; not identified [12]	[11, 12]
LNK p.E208Q (PH)	ET	JAK2 V617F [11]; not identified [12]	[11, 12]
LNK p.E208Q (PH)	PV (two patients)	JAK2 V617F	[13]

Polycythemia vera (PV) or essential thrombocythemia (ET) or primary myelofibrosis (PMF) or MDS/MPN with ring sideroblasts and thrombocytosis.

## Data Availability

The data analyzed in the current work are available from the corresponding author on reasonable request.

## Supplementary Materials

Supplementary Materials Serial values of complete blood count with relevant white blood cell differential counts are provided, showing the changes over time since her original diagnosis of multiple sclerosis till the diagnosis of CMML and beyond.
