# Supplementary material for: The Missing LNK: Evolution from Cytosis to Chronic Myelomonocytic Leukemia in a Patient with Multiple Sclerosis and Germline SH2B3 Mutation
Source: Case Rep Genet. 2022 Mar 1;2022:6977041. doi: 10.1155/2022/6977041 (PMC8904908; doi:10.1155/2022/6977041)
Supplement: Supplementary Materials — Serial values of complete blood count with relevant white blood cell differential counts are provided, showing the changes over time since her original diagnosis of multiple sclerosis till the diagnosis of CMML and beyond. [file 6977041.f1.docx]

**Hematologic changes over time**

|  | **At the diagnosis of MS** | **At first bone marrow biopsy (20 months later)** | **At second bone marrow biopsy (44 months later)** | **57 months later (Current)** |
| --- | --- | --- | --- | --- |
| White blood counts (4,000-11,000/μL) | 17,600 | 17,700 | 19,800 | 21,000 |
| Hemoglobin  (12-16 g/dL) | 15.5 | 14.9 | 14 | 14.4 |
| Mean Corpuscular Volume (80-100 fL) | 86.7 | 87.3 | 83 | 81.6 |
| Platelet count  (150,000-400,000/μL) | 407,000 | 457,000 | 440,000 | 499 |
| Absolute Neutrophil count (1300-7500/μL) | 11,800 | 11,300 | 14,000 | 14,600 |
| Absolute lymphocyte count (700-3900/μL) | 4100 | 4600 | 3300 | 4100 |
| Absolute monocyte count (100-1000/μL) | 1300 | 1300 | 2000 | 1900 |
